# The Two-Domain LysX Protein of *Mycobacterium tuberculosis* Is Required for Production of Lysinylated Phosphatidylglycerol and Resistance to Cationic Antimicrobial Peptides

**DOI:** 10.1371/journal.ppat.1000534

**Published:** 2009-07-31

**Authors:** Erin Maloney, Dorota Stankowska, Jian Zhang, Marek Fol, Qi-Jian Cheng, Shichun Lun, William R. Bishai, Malini Rajagopalan, Delphi Chatterjee, Murty V. Madiraju

**Affiliations:** 1 Department of Biochemistry, The University of Texas Health Center at Tyler, Tyler, Texas, United States of America; 2 Department of Microbiology, Immunology and Pathology, Colorado State University, Fort Collins, Colorado, United States of America; 3 Department of Medicine; Center for Tuberculosis Research, Johns Hopkins University School of Medicine, Baltimore, Maryland, United States of America; Harvard School of Public Health, United States of America

## Abstract

The well-recognized phospholipids (PLs) of *Mycobacterium tuberculosis* (*Mtb*) include several acidic species such as phosphatidylglycerol (PG), cardiolipin, phosphatidylinositol and its mannoside derivatives, in addition to a single basic species, phosphatidylethanolamine. Here we demonstrate that an additional basic PL, lysinylated PG (L-PG), is a component of the PLs of *Mtb* H37Rv and that the *lysX* gene encoding the two-domain lysyl-transferase (*mpr*F)-lysyl-tRNA synthetase (*lys*U) protein is responsible for L-PG production. The *Mtb lysX* mutant is sensitive to cationic antibiotics and peptides, shows increased association with lysosome-associated membrane protein–positive vesicles, and it exhibits altered membrane potential compared to wild type. A *lysX* complementing strain expressing the intact *lysX* gene, but not one expressing *mprF* alone, restored the production of L-PG and rescued the *lysX* mutant phenotypes, indicating that the expression of both proteins is required for LysX function. The *lysX* mutant also showed defective growth in mouse and guinea pig lungs and showed reduced pathology relative to wild type, indicating that LysX activity is required for full virulence. Together, our results suggest that LysX-mediated production of L-PG is necessary for the maintenance of optimal membrane integrity and for survival of the pathogen upon infection.

## Author Summary

The human pathogen *Mycobacterium tuberculosis* (*Mtb*) survives in the hostile intracellular environment, in part, by withstanding the actions of host-induced cationic antimicrobial peptides (CAMPs). Membrane phospholipid composition and the resultant charge could play an important role in *Mtb* survival within the host. Acidic phospholipids such as cardiolipin, phosphatidylinositol and its mannoside derivatives, phosphatidylglycerol, and a single basic species, phosphatidylethanolamine, are constituents of the *Mtb* membrane bilayer. We demonstrate that lysinylated phosphatidylglycerol (L-PG) represents another basic phospholipid and that the *lysX* gene, which encodes a two-domain protein with lysyl transferase and lysyl-tRNA synthase activities, is necessary for L-PG production. We show that L-PG is required for maintenance of an optimal membrane potential and resistance towards CAMPs. Phagosomes containing the *lysX* mutant showed an increased association with lysosomes, and the *lysX* mutant showed growth defects in mouse and guinea pig lungs, indicating that LysX activity is required for full virulence. Collectively, our results suggest that LysX activity, which is responsible for the production of L-PG, is necessary for maintenance of an optimal membrane potential such that the pathogen can grow optimally upon infection, presumably by withstanding the actions of CAMPs.

## Introduction

*Mycobacterium tuberculosis* (*Mtb*), the causative agent of tuberculosis, is a successful human pathogen that has infected more than one-third of the world's population. The success of *Mtb* as an infectious agent relies, in part, on its ability to modulate the expression of bacterial factors in response to infection so that it can successfully multiply within the hostile host environment [1]. The characteristic lipid-rich cell envelope of *Mtb* is one of the factors believed to contribute to its survival in vivo [2],[3]. It is generally believed that *Mtb* polar lipids (PoLs) consisting of acidic phospholipids (PL) such as cardiolipin (CL), phosphatidylglycerol (PG), phosphatidylinositol and its mannoside derivatives, in addition to basic PL such as phosphatidylethanolamine, are important constituents of the *Mtb* membrane [2]. *Mtb* PLs are known to function as important immune modulators [4] and have been shown to be released within phagosomes and transferred into lysosomes [5],[6]. It is interesting to note that PG, which is an abundant PL in other bacteria, is only a minor species in Mycobacteria, whereas CL is a major species [2],[3] with a high turnover rate [7].

The relative ratio of acidic to basic PLs is one of the determinants of net membrane charge. In some Gram-positive pathogens such as *Staphylococcus aureus* and *Listeria monocytogenes*, a fraction of the PG or CL molecules, or both, are lysinylated by the esterification of a glycerol hydroxyl group to lysine. Lysinylation imparts a net positive charge to these acidic PLs. This could, in turn, influence the ratio of acidic to basic PLs, resulting in an altered membrane charge. This could explain the bacterial susceptibility to cationic antibiotics (CAMAs) and peptides (CAMPs) [8],[9]. Although *Mtb* PLs have been well characterized for more than four decades, it is unknown if lysinylated PLs are a subset of the *Mtb* PLs and, if so, what the consequences associated with the absence of these lysinylated PLs might be. The present study demonstrates that the *Mtb lysX* gene, encoding a two-domain protein, is required for the production of lysinylated PG (L-PG), and the absence of L-PG is associated with changes in membrane potential, increased sensitivity to CAMAs and CAMPs, and growth defects in vivo.

## Results

### Identification of Lysinylated PLs in *Mtb*

**Figure 1:**
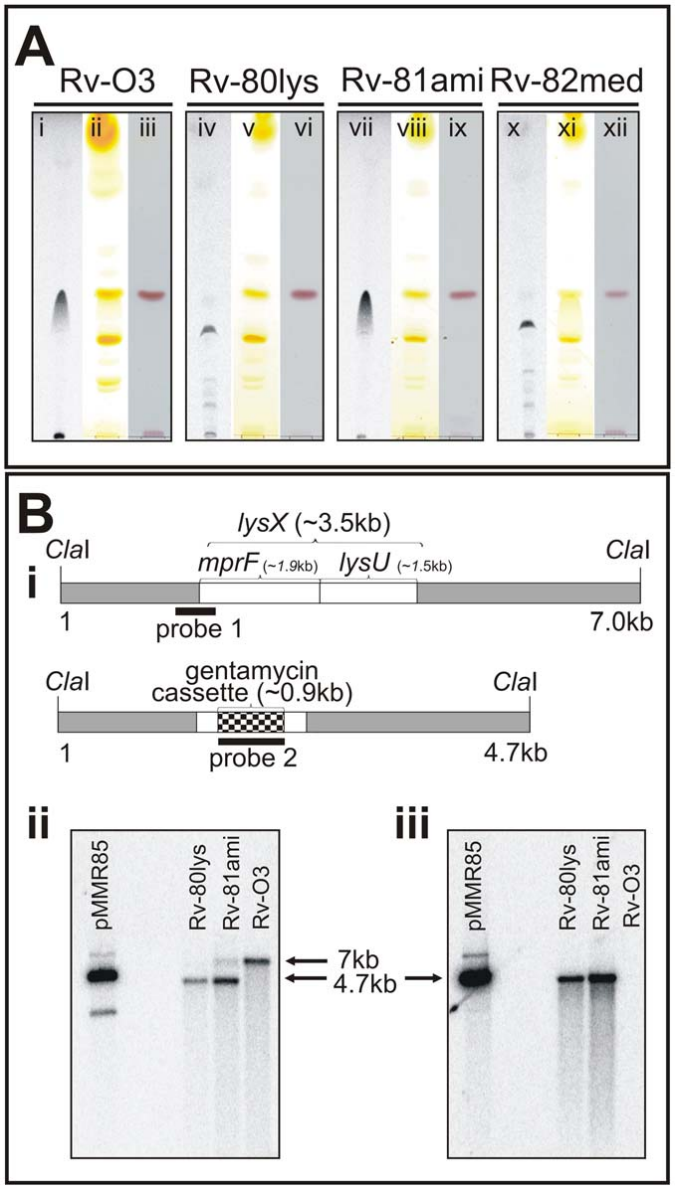
Polar lipid and Southern blot analysis of the *lysX* mutant strain. A: *Mtb* strains were grown in the presence and absence of ^14^C-lysine. Total lipids were extracted in chloroform∶methanol (2∶1 v/v) and resolved by TLC on Silcia Gel 60 (EMD Chemicals, New Jersey) in a solvent system of chloroform∶methanol∶water (65∶25∶4 v/v/v). TLC plates were either visualized by autoradiography (lanes i, iv, vii and x), exposed to iodine vapors (lanes ii, v, viii and xi), or stained with ninhydrin (lanes iii, vi, ix and xii). B: Southern blot analysis of *Mtb lysX* mutant strains. B-i: The *Cla*I fragment bearing the wild type *lysX* gene (3.5 kb) with the locations of the *mprF* and *lysU* regions marked. The dark box designated as “probe 1” is an approximately 750 bp fragment that hybridizes with the 5′-end of *lysX* and 160 bp of the *lysX* coding region. The *Cla*I fragment bearing the mutant *lysX* gene disrupted with the gentamycin cassette (0.9 kb) is also shown. The dark band designated as “probe 2” is the 900 bp gentamycin gene that hybridizes with the mutant *lysX* gene. B-ii: Southern blot analysis of *Cla*I-digested *Mtb* genomic DNA hybridized with probe 1. The 7 kb and 4 kb band positions represent Rv-03 and Rv-80lys, respectively. Note that the complemented copy contains a band corresponding to the integrated copy of *lysX* gene plus the flanking plasmid sequence. B-iii: Southern blot analysis of *Cla*I-digested *Mtb* genomic DNA (see Fig. 1B-iii) hybridized with probe 2. pMMR85 is a positive control plasmid containing the mutant *lysX* gene plus flanking regions.

In order to detect lysinylated PLs in *Mtb*, actively growing cultures were incubated with ^14^C-lysine for 3 days; total lipids were extracted, and PoLs were resolved by thin layer chromatography (TLC). A distinct radiolabeled lipid was evident, indicating that lysinylated PLs are members of the *Mtb* PoL pool (Fig. 1A, lane i). In *S. aureus*, the *mprF* gene is responsible for L-PG production [8]. Homology searches of the *Mtb* genome identified Rv1640c as *lysX*, which encodes an *mprF*-like gene as a fusion to a lysyl-tRNA synthetase (*lys*U). The latter gene is distinct from the essential housekeeping tRNA synthetase (Rv3598c). The *mprF* gene in *S. aureus* encodes a protein with potential lysyl transferase activity [10]. In order to evaluate the function of *lysX*, we created a *lysX* mutant strain, Rv-80lys, by replacing the majority of the coding region comprising the *mpr*F and *lys*U domains with a gentamycin resistance cassette using homologous recombination (see Methods section). A complementing derivative of this strain, Rv-81ami, was created by integrating a plasmid expressing the intact *lysX* gene under the control of the *amidase* promoter [11]. The *lysX* mutant strain was found to be defective in the production of L-PoLs (Fig. 1A, lane iv compared with lane i). L-PoL production was restored, however, in the *lysX* complemented strain Rv-81ami (Fig. 1A, see lane vii), confirming that the *lysX* gene product is responsible for the production of L-PoLs. Staining TLC plates with iodine (lanes ii, v, viii and xi) or ninhydrin (lanes iii, vi, ix and xii), on the other hand, did not detect L-PoLs, indicating that they may not be an abundant lipid species. We cultured *Mtb* in the presence of ^14^C-acetic acid and extracted total lipids, followed by TLC separation and subsequent quantification of L-PoL relative to total input radioactivity, and found that L-PoL accounts for approximately 0.3% of the total lipids (data not shown).

### Structural analysis of the lysinylated polar lipid

**Figure 2:**
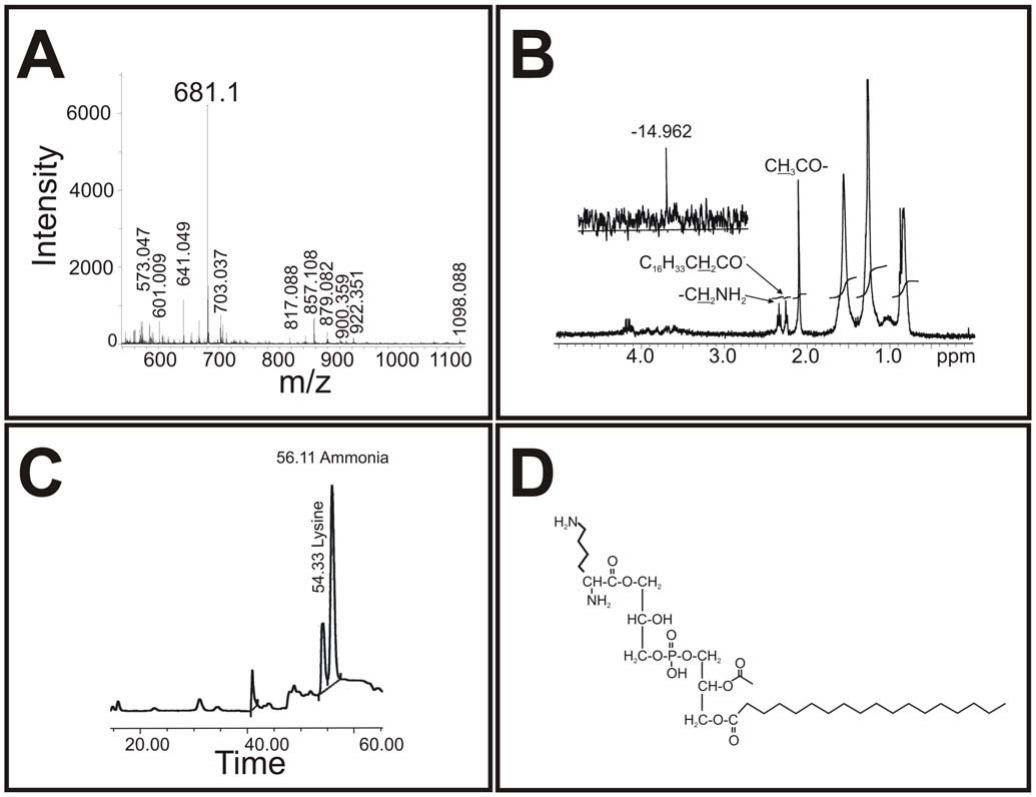
Structural analysis of L-PoL. A: MALDI mass spectrometry analysis of L-PG in negative-ion mode. The *m/z* 681 [M-H]^−^ represented the molecular ion peak. B: Structural analysis of L-PG. ^1^H-NMR spectrum of L-PG. The acetyl group can be found at δ_CH3_ 2.1 ppm, and δ_CH2_ from the primary amine in lysine was detectable at 2.4 ppm. The inset shows the ^31^P-NMR spectrum, in which the phosphorus resonance shifted at −14.96 ppm. C: The amino acid profile of L-PG. Pure lipid was hydrolyzed with 6 N HCl for 24 h, and the soluble hydrolytic product was analyzed in an amino acid analyzer. Note that the elution of lysine at 54.33 min coincides with the standard (not shown). The small peak at position 41 min corresponds to a buffer change during the run, and the ammonia peak at 56.11 min is from the buffer used to run the analyzer. D: The proposed L-PG structure. The proposed structure of L-PG with a C_18_ fatty acid identified from fatty acid analysis (data not shown).

In order to determine the nature of the L-PoL, preparative 2D-TLC was carried out to collect L-PoLs. Structural analysis of the L-PoL was carried out using a combination of MALDI-MS, amino acid analysis and NMR (Fig. 2A–D). The MALDI-MS analysis in negative-ion mode revealed *m/z* 681 ([M-H]^−^) to be the molecular ion peak (Fig. 2A). The ^1^H-NMR results confirmed the presence of an acetyl group at δCH_3_ 2.1 ppm and δCH_2_ from the primary amine in lysine at δ 2.4 ppm (Fig. 2B), whereas the ^31^P-NMR spectrum showed a shift in the phosphorus resonance spectrum at δ −14.96 ppm (Fig. 2B inset). Fatty acid analysis demonstrated that the molecule was C18 (data not shown), and amino acid analysis following acid hydrolysis confirmed the presence of lysine (Fig. 2C). Together, these data demonstrate that lysine is covalently linked to PG with the predicted structure shown in Fig. 2D. The L-PoL in the text is referred to hereafter as L-PG. Similar structural analyses of the corresponding unlabeled PoL of the slower migrating radioactive lipid species of *lysX* (Fig. 1A, lane iv) could not be done, given that it was present in negligible quantities (not shown). The thermal decomposition products of lysine are well characterized [12]. We speculate that the PoL accumulation in the *lysX* strain is a consequence of lysine degradation. Further studies are required to clarify the nature of the lipid species accumulating in the *lysX* mutant.

### An intact *lysX* gene containing *lys*U and *mpr*F domains is necessary for the production of L-PG

As previously noted, the *Mtb lysX* is a fusion gene encoding both *mpr*F and *lys*U activities, with *mprF* located at the 5′ end of the *lysX* gene (see Fig. 1B). The Gram-positive bacteria that have been shown to produce L-PG, however, contain only *mprF*. The *Mtb* MprF and *S. aureus* MprF share three domains of unknown function, DUF470, DUF471 and DUF472. In order to evaluate whether L-PG production in *Mtb* requires the activities of both the LysU and MprF domains, we generated Rv-82med, a *lysX* complemented derivative that produces only the MprF domain (see Methods) and evaluated its ability to produce L-PG following the incubation of actively growing cells with radiolabeled lysine. The Rv-82med strain, much like Rv-80lys, was defective in L-PG production (see Fig. 1A, lane x and compare with lane iv). Quantitative real-time PCR analysis using primers and TaqMan probes targeted to the *mprF* region of *lysX* (compared to the 16S rRNA housekeeping gene) revealed that the expression of *mprF* in Rv-82med was comparable to that in Rv-03 wild type and Rv-81ami (data not shown). Together, these results indicate that Rv-82med expresses *mprF* and that the MprF domain alone is not sufficient for the production of L-PG in *Mtb*.

### Phenotypes associated with the absence of L-PG production

**Figure 3:**
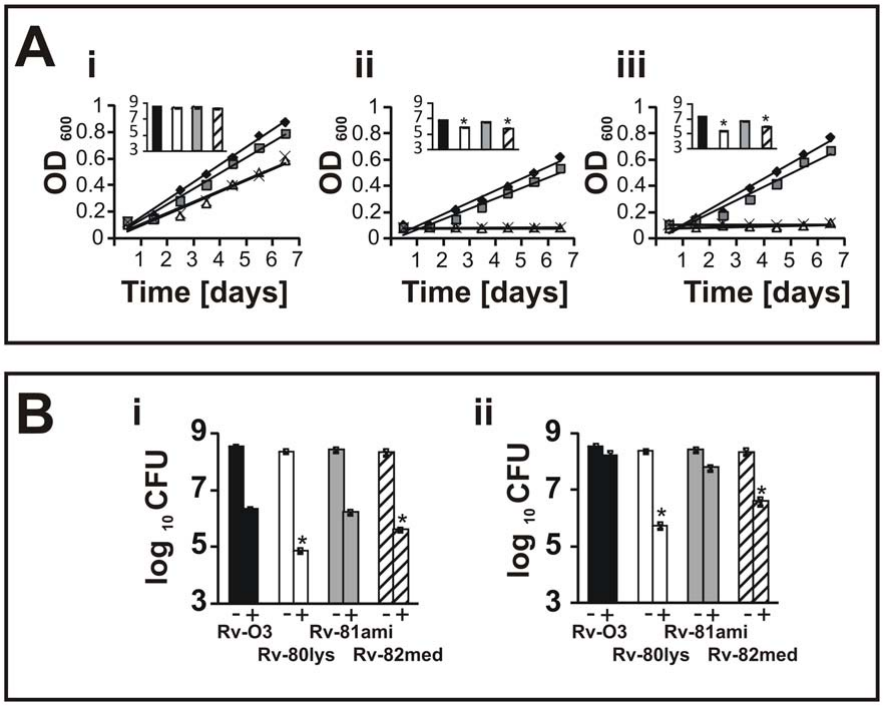
Phenotype of *lysX* strains. Panel A: The growth and viability of the *Mtb lysX* strains in 7H9 broth in the absence of antibiotics -i. A-ii: The growth and viability of cultures grown in the presence of 1.0 µg/ml Van. At the indicated times, growth was measured. After 3 days of growth in broth, viability was determined by plating cells on Middlebrook 7H11 agar and determining the CFU. Symbols: Filled diamonds – Rv-03; grey squares – Rv81-ami; white triangles – Rv-80lys; crosses – Rv-82med. The inset shows the viable cell count. Black bars - Rv-03, grey bars - Rv-81ami, white bars - Rv-80lys and dashed bars - Rv82-med. A-iii: The growth and viability of cultures grown in the presence of 100 units/ml PMB. * Represents a *P* value<0.001 versus Rv-03 and Rv-81ami (Student-Newman-Keuls Method); bars represent means±standard error. Panel B: The growth and viability of *Mtb* strains in the presence of 1 mg/ml lysozyme -i- or HNP-1 -ii. There was no significant reduction in viability compared to cultures grown without TFA (data not shown). The stars represent P<0.001 versus Rv-03 and Rv-81ami (Student-Newman-Keuls Method). The bars represent the mean±standard error.

Gram-positive organisms such as *S. aureus* and *B. subtilis* are sensitive to cationic antimicrobial antibiotics (CAMAs) such as vancomycin (Van) and polymyxin-B (PMB) and to cationic antimicrobial peptides (CAMPs) such as human neutrophil peptide (HNP-1) and lysozyme. On the other hand, *Mtb* is generally tolerant to these compounds. HNP-1 and lysozyme are produced in neutrophils and macrophages, respectively. It is generally believed that CAMPs induce cell death by interfering with the integrity of the negatively charged membrane. Furthermore, the ability of intracellular pathogens to resist the action of CAMPs produced by the host is, in part, responsible for pathogen proliferation upon infection [13]. The presence of lysine groups on the acidic PG would impart a net positive charge and, therefore, could affect the net ratio of positively charged to negatively charged PL species. Thus, the absence of L-PG could make the *Mtb* membrane relatively acidic, thereby sensitizing the bacterium to the action of CAMAs. To test this possibility, actively growing Rv-03, Rv-80lys, Rv-80ami and Rv-82med were exposed to Van and PMB, and growth and viability were measured (Fig. 3A). Van and PMB interfered with the growth and viability of Rv-80lys and Rv-82med (see Fig. 3A-ii and 3A-iii), inset showing viability after 3 days of exposure; and Figure S1 showing viability after 6 days of exposure). Comparisons of growth, measured as the change in optical density (OD), and viability, measured as the change in CFU, revealed that while the *lysX* mutant was relatively more sensitive to Van and PMB than Rv-03, it was able to recover when grown in the absence of antibiotics, indicating that Van and PMB do not exert potent bactericidal activity. All of the strains grew well in the absence of antibiotics, although the *lysX* mutant showed a small reduction in growth rate in the absence of antibiotics (Fig. 3A-i), inset shows an approximately 0.3 log reduction in viability). Visualization of Rv-80lys cells following nucleoid staining and bright field or fluorescence microscopy did not reveal any significant differences in cell morphology or nucleoid organization (data not shown).

The increased sensitivity of the *lysX* strain to antibiotics suggests an enhanced association between the two. To test this possibility, actively growing *lysX* and Rv-03 cells were stained with fluorescent-vancomycin (Fl-Van), and the staining patterns were visualized by fluorescence microscopy. Earlier studies revealed that in stained *Mtb* cells, Fl-Van associates with the nascent growth zones, primarily at the poles and mid-cell septa [14]. These studies also indicated that not all *Mtb* cells could be stained with Fl-Van [14]. We found that a higher percentage of *lysX* cells were stained with Fl-Van compared to Rv-03 (see Figure S2). Approximately 52% of *lysX* cells showed staining patterns not only at the mid-cell and polar septa but also over the entire length; meanwhile, only 32% of wild type cells showed such a staining pattern (see Figure S2 legend for details). These results are consistent with the idea that Van is able to gain better access to Rv-80lys cells compared to Rv-03 cells.

Next, we examined whether the *lysX* mutant was also sensitive to lysozyme and HNP-1. Similar to the results seen with the CAMAs, HNP-1 and lysozyme significantly reduced the viability of Rv-80lys compared to Rv-03 and Rv-81ami (Fig. 3B-i and 3B-ii), see legends for *P* values). The phenotype of Rv-82med was found to be similar to that of Rv80lys (Fig. 3B). Together, these results indicate that the absence of L-PG production is associated with increased sensitivity of the bacterium to the actions of CAMPs and CAMAs. Importantly, these experiments also showed that complementation of the *lysX* mutant restored the wild type growth phenotype under these conditions (Fig. 3A and 3B).

### Altered membrane potential of *Mtb lysX* mutants

**Figure 4:**
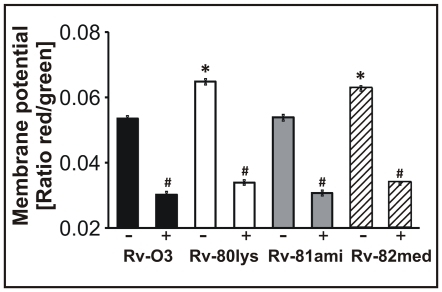
Determination of the membrane potential of *Mtb* strains. The relative membrane potential was calculated using the average mean fluorescence intensity (the ratio between the average red fluorescence and the average green fluorescence). The graph shows the average of four experiments. Wild type and *lysX* mutant strains were incubated with 3 µM DiOC_2_(3) for 5 h in either the presence (+) or absence (−) of 100 µM CCCP. The bars represent the mean±standard error. * Represents *P*<0.002 versus Rv-03 and Rv-81ami (Student-Newman-Keuls Method), whereas # refers to *P* = 0.001 compared to untreated with CCCP.

We wished to test whether the absence of L-PG production in Rv-80lys cells was associated with changes in the properties of the PL bilayer (e.g., membrane potential). The membrane potential of the Rv-80lys cells was determined using a slow-response membrane potential-sensitive dye, DiOC_2_(3), and comparing with Rv-03 cells. This cationic cyanine dye exhibits green fluorescence (*Ex* = 488 nm and *Em* = 520 nm) in the monomeric state and red fluorescence (*Ex* = 488 nm and *Em* = 620 nm) in the aggregated or oligomeric state. As a negative control, the membrane potential was measured following exposure of the cells to the proton ionophore *m*-chlorophenylhydrazone (CCCP), which is known to eliminate the proton gradient across the membrane. As seen in Figure 4, the membrane potentials (measured as the ratio of red to green fluorescence) of the *lysX* mutant Rv-80lys and Rv-82med were 21% and 17%, respectively, higher than that of the Rv-03 and complemented Rv-81ami (*P*<0.002). The increased ratio of red to green fluorescence observed in *lysX* mutants suggests accumulation of the positively charged lipophilic dye on the negatively charged membrane. The red to green fluorescence ratio in all strains was decreased to similar levels (∼41%) in the presence of CCCP (*P* = 0.001). Presumably, this reduction reflects the completely depolarized state of the membrane. Together, these results indicate that the membranes of Rv-80lys and Rv-82med are hyperpolarized relative to Rv-03 and Rv-81ami.

### *lysX* mutant phenotype in macrophages

**Figure 5:**
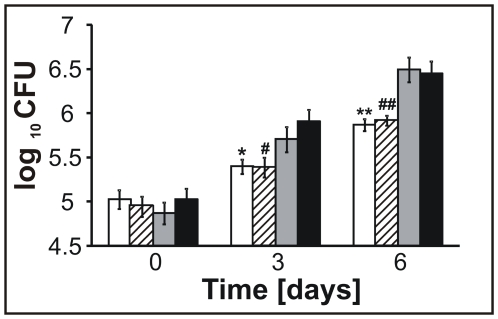
The viability of *lysX* in macrophages. THP-1-derived macrophages were infected with *Mtb* strains; at the indicated times following infection, the macrophages were lysed, and the *Mtb* viability was determined. The white bars represent Rv-80lys; the dashed bars represent Rv-82med; the grey bars represent Rv-81ami; and the black bars represent Rv-03. Data are mean±standard error from three independent experiments, and the Mann-Whitney Rank Sum Test was used for data analyses.

We next examined whether the *Mtb lysX* mutant Rv-80lys showed proficient growth in macrophages upon infection of the THP-1 macrophage cell line. The Rv-80lys showed a modest growth defect in macrophages compared to Rv-03 and complemented Rv-81ami (see Fig. 5, *P* = 0.01 for day 3, *P* = 0.006 for day 6). Similar results were also noted for Rv-82med (Fig. 5, *P*<0.014 for day 3 and *P*<0.03 for day 6 compared to Rv-03).

**Figure 6:**
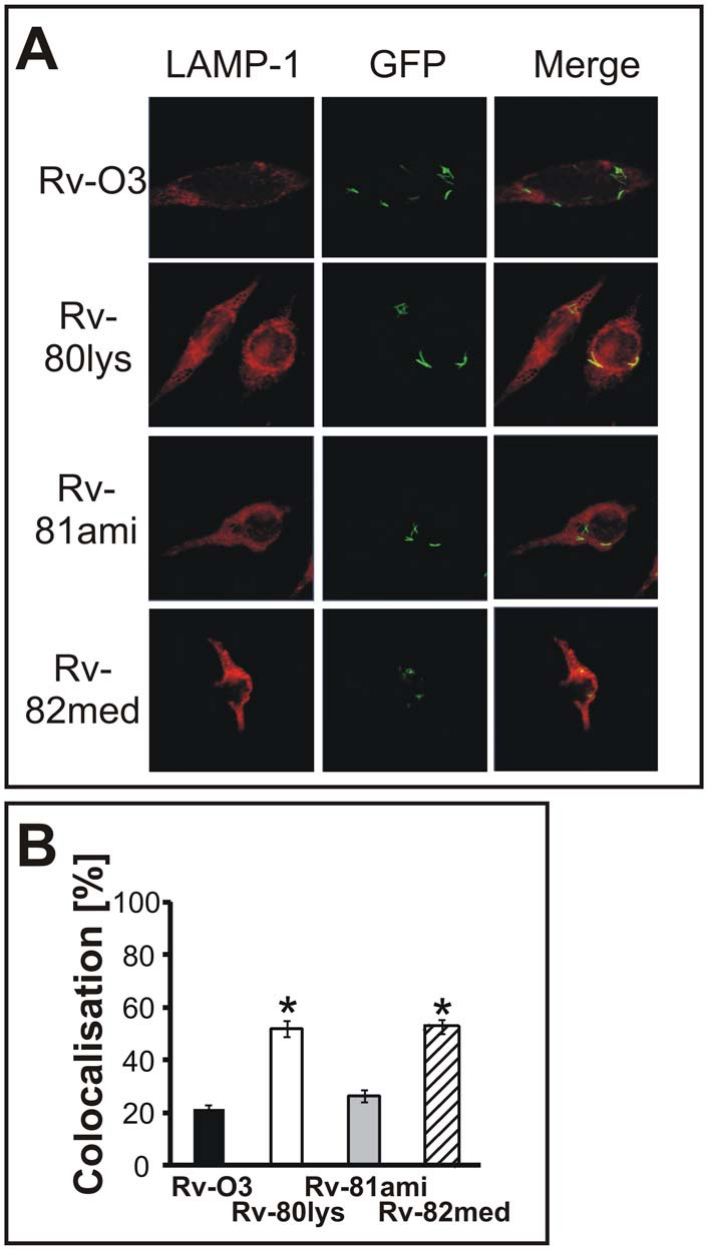
Co-localization of *Mtb* with LAMP-1-expressing phagosomes. Panel A: THP-1-derived macrophages were infected with GFP-expressing *Mtb* strains (Rv-03, Rv-80lys, Rv-81ami and Rv-82med). Bacteria (green spots) inside LAMP-1-positive phagosomes (red) produce a yellow signal indicating co-localization (merged). Panel B: The percent co-localization was determined by visual scoring of yellow spots after 72 h of infection. We analyzed 126 macrophages for Rv-80lys, 164 for wild type, 168 for complemented, and 127 for Rv-82med and scored 534 bacterial cells for Rv-80lys, 1,307 for wild type, 848 for Rv-81ami, and 402 for Rv-82med. *Mtb* Rv-03 (black bar; 21.2±1.6%), Rv-80lys (white bar; 52.0±3.0%), Rv-81ami (grey bar; 26.4±2.2%) and Rv-82med (dashed bar; 52.6±2.8%). * *P*<0.001 versus Rv-03 and Rv-81ami using the Student-Newman-Keuls Method; bars represent mean±standard error.

Intracellular replication of *Mtb* is, in part, due to its ability to resist the delivery of its phagosomes to lysosomes [15]. This process can be visualized by examining the co-localization of *Mtb* with the lysosome-associated-membrane protein (LAMP-1). In order to address whether the *lysX* mutant had a phagosome-lysosome fusion defect, we infected macrophages with *Mtb* strains expressing green-fluorescent protein and visualized co-localization with LAMP-1. Increased association of phagosomes containing Rv-80lys with lysosomes was evident compared to Rv-03 and complemented Rv-81ami (Fig. 6, *p*<0.001). Rv-82med behaved like Rv-80lys, indicating that the full-length *lysX* gene is required for functional activity. These results are consistent with the hypothesis that the *lysX* mutant is not as proficient as the Rv-03 strain in preventing fusion of phagosomes with lysosomes, which could contribute to defects in intramacrophage replication.

**Figure 7:**
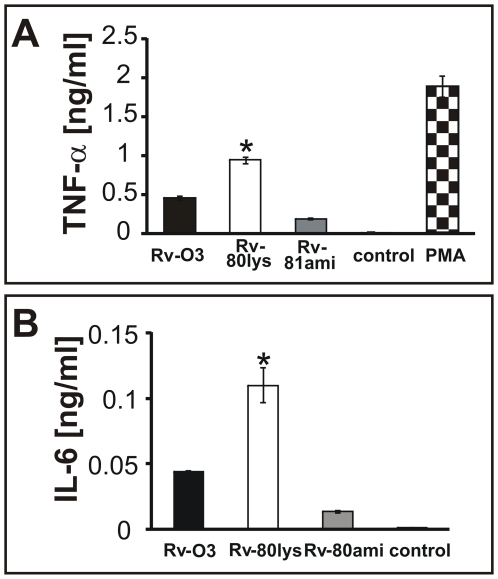
Select cytokine responses of macrophages. Panel A: MDMO were infected with *Mtb* strains, and TNF-α release was measured by ELISA. PMA was used as a positive control. TNF-α levels in panel A were measured after 24 h. * Represents *P*<0.05 compared to Rv-03, Rv-81ami, control (untreated cells) and PMA. Panel B: Secretion of IL-6 by MDMO after 48 h of infection. * *P*<0.05 compared to Rv-03, Rv-81ami and control. The experiments were done in duplicate, and representative results are shown. Data are mean±standard error. Student-Newman-Keuls Method was used.

The production of inflammatory cytokines tumor necrosis factor-alpha (TNF-α), IL-6 and IL-10 is necessary to mount a protective immune response against *Mtb* infection [16],[17],[18]. TNF-alpha restricts the growth of *Mtb* in alveolar macrophages [18], and the multiplication of virulent *Mtb* in monocyte-derived macrophages (MDMO) is associated with suppression of TNF-α production during the early periods after infection. To test selected pro-inflammatory cytokine responses of macrophages, MDMO were infected with Rv-80lys and Rv-03 strains, and the production of TNF-α and IL-6 was measured (Fig. 7). As can be seen, the secretion of TNF-α was elevated after infection with the *lysX* mutant compared to the wild type and complemented strains, similar to MDMO cells exposed to PMA (Fig. 7A). Similarly, the secretion of IL-6 was also increased following infection with the *lysX* mutant compared to the wild type and complemented strains (Fig. 7B).

### *lysX* has growth defects in vivo

**Figure 8:**
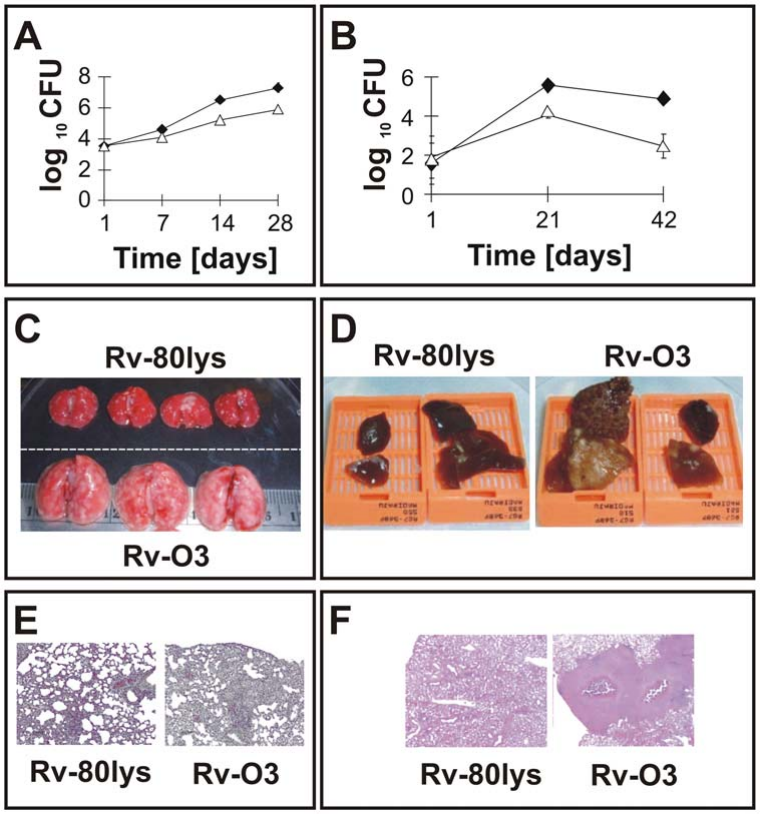
Growth of *lysX* and WT strains in vivo. Panel A: C57BL/6 female mouse lungs were infected with *lysX* (triangles) and WT (dark diamonds) strains via an aerosol route. The mean CFU counts were obtained from lung homogenates of at least three mice per group and plated on Middlebrook 7H10 agar plates. All plates were incubated at 37°C for at least three weeks before colonies were counted. Panel B: Guinea pigs (Hartley strain) were infected with *lysX* (open symbols) or WT (dark symbols) strains via an aerosol route. Five guinea pigs were sacrificed at days 1, 21 and 42 days after infection, and the survival of *Mtb* strains in the entire lung homogenate was determined by plating on agar medium as described above. Panels C, D: Gross pathology of the lungs infected with the Rv-80lys and Rv-03 *Mtb* strains. Lungs from mouse -C- and guinea pig -D- were excised, stored in 10% formalin, embedded, and stained with hematoxylin and eosin for histopathological analysis. Note the presence of tubercles on the surface of the lungs for Rv-03 compared to Rv-80lys. Panels E, F: Histopathology of mouse -E- and guinea pig -F- lungs infected with the Rv-80lys and Rv-03 *Mtb* strains. Hematoxylin-eosin staining confirmed that the lungs infected with Rv-03, but not those infected with Rv-80lys, had extensive inflammation in both species and showed caseating granulomas in guinea pigs.

To evaluate the phenotype of *lysX* in vivo, C57BL/6 mice and Hartley strains of guinea pigs were aerosol infected with *lysX*, and the viability of the pathogen was measured (Fig. 8). The *lysX* mutant showed only a modest growth defect in mice (Fig. 8A) but was clearly attenuated in guinea pigs (Fig. 8B) and showed reduced dissemination to the spleen (Figure S4). Gross pathology and histopathology of the lungs of infected mice at 28 days (Fig. 8C and E) and guinea pigs at 42 days (Fig. 8D and F) showed distinct differences between the wild type and the *lysX* mutant. Hematoxylin-eosin staining confirmed that the lungs infected with Rv-03, but not those infected with Rv-80lys, had extensive inflammation in both species and showed caseating granulomas in guinea pigs. The vast differences in the growth kinetics and pathology between the wild type and *lysX* mutant pathogens indicate that LysX activity is required for full virulence. Interestingly, the *lysX* complemented strain Rv-81ami behaved like the *lysX* mutant with respect to in vivo growth and pathology (data not shown), indicating that the complemented strain is not able to restore *lysX* function in vivo.

The ability of *Mtb* strains to produce complex cell wall-associated lipids called phthiocerol dimycocerosates (PDIM) and to bind and reduce neutral red dye is associated with virulence. Avirulent and attenuated strains are defective in these processes. Furthermore, virulent *Mtb* strains propagated in the laboratory often lose these properties [2],[19],[20],[21]. The neutral red reduction and PDIM profiles of the *lysX* mutant were comparable to those of wild type cells (Figure S5), indicating that the observed in vivo growth defects of the *lysX* mutant are not due to a loss of PDIM and defect in neutral red reduction.

## Discussion

The primary conclusion of our data is that L-PG is one of the basic PLs in *Mtb* and that the *lysX* gene, encoding the two-domain LysX protein, is responsible for its production. Although L-PG is a minor PL species of *Mtb*, its absence has several consequences, one of which is an alteration of the membrane potential. This underscores the role of LysX activity in maintaining optimal membrane function. Presumably, the absence of L-PG in the *lysX* mutant shifts the ratio of acidic to basic PLs, thereby hyperpolarizing the membrane. A consequence of the absence of L-PG is the increased sensitivity of the pathogen to lipophilic antibiotics such as PMB and Van. It is likely that hyperpolarization of the membrane in the *lysX* mutants due to its net negative charge promotes interactions with cationic peptides and antibiotics produced by the host immune system, which in turn could lead to the killing of the invading pathogens [13],[22]. It is known that host-induced CAMPs are one of the frontline defenses against invading pathogens. Therefore, sensitization of *lysX* mutant *Mtb* cells to the action of CAMPs suggests that maintenance of the optimal membrane potential is necessary for *Mtb* growth in vivo.

**Figure 9:**
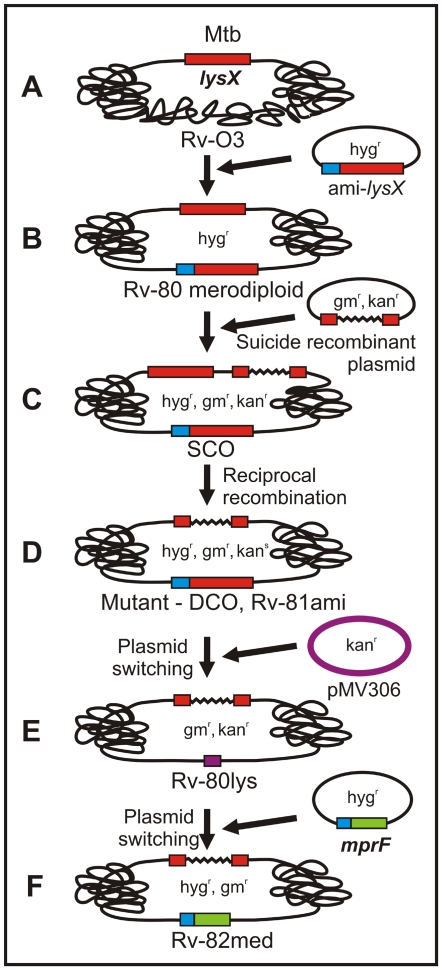
Cartoon showing construction of the *lysX* mutant and complemented strains. A- refers to the wild type strain carrying the *lysX* gene at its native locus. B- refers to the Rv-80 merodiploid (hyg^r^) strain produced by the integration of a plasmid expressing the *lysX* gene from the amidase promoter. C- refers to a single crossover (SCO, hyg^r^, kan^r^, gm^r^) recombinant produced by integration of the suicide recombination plasmid. D- refers to a mutant double crossover (DCO, hyg^r^, kan^s^, gm^r^) produced following a reciprocal recombination event. E- refers to Rv-80lys (kan^r^, gm^r^) produced following switching of the resident integrated plasmid (hyg^r^) with the incoming pMV306 plasmid (kan^r^). F- refers to Rv-82med (hyg^r^, gm^r^) produced following switching of the resident pMV306 plasmid (kan^r^) with the incoming plasmid expressing the *mprF* fragment from the amidase promoter (hyg^r^).

In partial support of this claim, we found that the *lysX* mutant showed defects in intracellular replication (Fig. 5) and that infection of macrophages with *lysX* led to increased production of pro-inflammatory cytokines (Fig. 7). We also found that the *lysX* mutant showed increased co-localization with LAMP-1 vesicles (Fig. 6). Finally, we showed that the *lysX* mutant was attenuated in guinea pig lungs and had a modest growth defect in mouse lungs (Fig. 9). Together, these results are consistent with the hypothesis that LysX activity is required to maintain an optimal membrane potential and possibly to promote pathogen survival upon infection. Notably, the gross pathological differences between the *lysX* mutant and wild type were striking compared to the modest differences in growth in vitro and *ex vivo* (see Figs. 3, 5 and 8). The reduced bacterial burden and the reduced pathology and size of granulomas in the lungs of guinea pigs clearly suggest that LysX activity is required for bacterial multiplication and virulence. Evaluation of the host-induced cytokine response following different stages of infection with wild type and *lysX* mutant pathogens could provide valuable insights into *lysX* function. Our studies also showed that the *lysX* mutant, like wild type, retained the ability to produce PDIMs and reduce neutral red (Fig. S5). It remains to be evaluated, however, if other membrane and cell wall-associated lipids are modulated in the *lysX* background.

The production of L-PG is believed to involve two biochemical steps: the generation of lysyl-tRNA by the LysU protein and the transfer of a lysine group from the lysyl-tRNA to PG by MprF, a membrane-bound lysyl-transferase protein [23]. The Gram-positive bacteria shown to produce L-PG carry a single housekeeping *lysU* gene that encodes a cytosolic LysU protein [8],[9],[24]. *E. coli* does not contain L-PG, but ectopic expression of the *S. aureus mprF* gene allows *E. coli* to accumulate L-PG in their membranes, suggesting that cytosolic LysU and membrane-bound MprF cooperate to produce L-PG [10],[25]. *Mtb* contains two *lysU* genes, one encoded by Rv3598c, which is an essential gene, and the other encoded by the *lys*U domain of *lysX* [26]. Since expression of the *mprF* fragment of *lysX* does not lead to the production of L-PG (Fig. 2), it appears that in *Mtb*, unlike in other bacteria, the cytosolic LysU and the membrane-bound MprF do not cooperate to produce L-PG.

This raises the question as to why a dedicated *lysU* gene product, distinct from the housekeeping gene, is required for L-PG production in *Mtb*. One possibility is that the lysinylation reaction occurs on the membrane, and the local presence of LysU and MprF activities are required to transfer lysine from the lysyl-tRNA to the membrane-bound PG. If the cytosolic lysyl-tRNA could not diffuse through the *Mtb* plasma membrane, a separate activity would be needed to replace it. Nonetheless, such dedicated activities imply that PG lysinylation in *Mtb* is a tightly regulated reaction. The temporal expression profile of *Mtb* genes upon infection in mice shows that *lysX* is upregulated during acute and chronic infection [27]. Presumably, increased expression levels of *lysX* would ensure that sufficient levels of L-PG were produced to maintain the optimal ratio of acidic to basic PLs. This would, in turn, ensure that the optimal membrane potential required for *Mtb* proliferation upon infection is maintained. Another possibility, although unlikely, is that the demand for lysyl-tRNA required for lysinylation and protein synthesis cannot be met by a single housekeeping enzyme. Clearly, however, further studies are required to address this issue.

While this manuscript was in preparation, Vandal et al. reported the characterization of several transposon mutants of *Mtb* that were hypersensitive to acidic pH, one of which was *lysX* [28],[29]. Their transposon mutants were hypersensitive to antibiotics and other stressors such as heat, SDS and DETA-NO. Although the *lysX* mutant was moderately sensitive to DETA-NO, its growth was not attenuated in murine lungs. It is unknown whether L-PG is produced in the *lysX* transposon mutant and whether the *lysX* mutant shows any residual activity. As shown in Fig. 1A, our *lysX* mutant was generated by removing most of the coding sequence responsible for producing the *mpr*F and *lys*U activities. We demonstrated that L-PG was not produced in the *lysX* mutant and that maintenance of the membrane potential and resistance to CAMPs were dependent on LysX activity. Importantly, we showed that LysX activity was required for full virulence in mice and guinea pigs. These results underscore the importance of *lysX* function in *Mtb* survival upon infection. One limitation of our results, however, is that the complemented Rv-81ami was not able to restore the *lysX* defect in vivo, although it did restore defects in other assays reported in this study. One possibility is that the expression of *lysX* in-trans at an *att*B locus was not sufficient to restore the LysX activity to optimal levels, and small changes in activity could have consequences for the complementation phenotype in vivo. Further studies are required to address this issue.

L-PG appears to be a minor lipid species, yet the loss of L-PG production affected membrane potential and *Mtb* growth in vitro and in vivo. It is interesting to note that PG, the purported substrate of L-PG, is also a minor lipid species in *Mtb* and other mycobacterial species [2],[3],[30],[31]. This raises the question of how the lysinylation of a minor PL species contributes to the observed phenotype. It is known that PG is a biosynthetic intermediate of CL, one of the major PL species of mycobacteria. Indeed, the enzymatic activities responsible for CL production from PG pools have been detected in mycobacteria [32]. PG also accumulates as a result of CL catabolism and, if unregulated, could be further processed to produce a diacylglycerol intermediate via the action of phospholipases [33],[34]. Accordingly, we speculate that the lysinylation step helps to prevent PG degradation such that the optimal membrane potential required for *Mtb* survival upon infection is maintained. Our results also suggest that changes in membrane potential are a potential mechanism for regulating CAMP sensitivity in *Mtb* and possibly in the *mprF* mutants of other bacteria; therefore, this could be exploited to develop novel antimicrobial compounds. It is tempting to speculate that by manipulating LysX activity, we could promote the action of other conventional antibiotics against *Mtb*.

## Methods

### Ethics statement

Mouse and guinea pig infection protocols were approved by the Animal Care and Use Committee at Johns Hopkins School of Medicine, Baltimore, MD for mice and at Texas A & M University, College Station, TX for guinea pigs, under the NIH contract (Tuberculosis Animal Research and Gene Evaluation Task force).

### Strains, media and culture conditions

The *Mtb* strains were cultured in Middlebrook 7H9 broth supplemented with 10% OADC (oleic acid, albumin, dextrose, catalase) and 0.05% Tween-80. As needed, 50 µg/ml hygromycin (hyg), 10 µg/ml kanamycin (kan) or 50 µg/ml gentamycin (gm) was added. For the determination of viable colonies and the scoring of recombinants, bacterial cells were plated on Middlebrook 7H11 agar plates containing the appropriate antibiotics. In some experiments, cultures were grown in the presence of L-[U-^14^C]-lysine (300 mCi/mmol, Amersham Pharmacia Biotech) or [1,2-^14^C] acetic acid (46 mCi/mmol, PerkinElmer), and total lipids were extracted and resolved by TLC. The radioactivity present in the L-PG spot was determined and normalized relative to total in put radioactivity.

### Construction of the *lysX* deletion and complemented derivative strains

The *lysX* coding region was cloned downstream of the *amidase* promoter in an integration-proficient, hygromycin-resistant plasmid and electrotransformed into *Mtb* in order to generate the *lysX* merodiploid strain. The chromosomal copy of the *lysX* gene was disrupted in the *lysX* merodiploid background by homologous recombination as described previously [35]. Using this approach, 90% of the *lysX* coding region was replaced with a 900-bp gentamycin resistance cassette. This strain, designated as Rv-81ami, was the *lysX* complemented strain. Next, the resident integrated plasmid encoding the functional *lysX* gene was replaced with an empty kanamycin-resistant plasmid to generate the *lys*X mutant strain, designated as Rv-80lys, as described [14],[35],[36],[37]. A cartoon depicting the *lys*X mutant and complemented strain construction is shown (Fig. 9). All strains were confirmed by PCR and Southern blot analysis.

For the generation of the *lysX* complemented strain expressing the *mpr*F domain, a 1,950 bp *lysX* gene encoding the *mpr*F domain was amplified by PCR using the primers MVM530lysF (5′-GGCGAATTCCATATGGGACTCCACTTAACTG-3′) and lys650MM606R (5′-AGC AGCAAGCTTCTAGAATCACGCCAACCGCTCGGGACTGC-3′) and cloned into the pJFR19 vector under the control of the *amidase* promoter [38]. The integrity of cloned insert was verified by DNA sequencing. This recombinant plasmid was used to replace the resident empty plasmid in the Rv-80lys mutant to generate Rv82-med. This strain was confirmed by PCR and Southern blot analysis.

### Intracellular growth measurements

The human monocyte cell line THP-1 (American Type Culture Collection, Rockville, Maryland) was used. Cells were grown in RPMI 1640 (Invitrogen, CA) supplemented with 2 mM L-glutamine, 1 mM sodium pyruvate, 10% fetal bovine serum (Invitrogen) and 100 U/ml penicillin G (Sigma, MO). The viability of the macrophages was determined using trypan blue staining. Monocytes were differentiated into macrophages by exposure to 50 nM PMA (phorbol 12-myristate 13-acetate; Sigma) and 7.5 ng/ml IFN-γ (human interferon-gamma, Peprotech) for 24 h, followed by a 24 h incubation with 50 nM PMA alone. The macrophages were washed three times with RPMI 1640 medium and incubated in medium that was not supplemented with PMA or IFN- γ for the next 24 h. Single cell suspensions of *Mtb* strains in RPMI 1640 media were used to infect 4.5×10^5^ macrophages in triplicate in a 24-well plate at a multiplicity of infection of 1∶2–4. After 3 hours of infection, macrophages were lysed in 0.09% SDS, and viability was determined to get a t0 count. No statistical differences in viability among these strains were noted at t0. Subsequently, macrophages at 3 and 6 days post-infection were also processed in order to determine *Mtb* viability. The *lysX* strains were no more sensitive than the other strains in terms of the concentrations of SDS used to lyse the macrophages and process them for viability determination (see Figure S3).

### Co-localization of *M. tuberculosis* with phagosomes expressing LAMP-1

THP-1 macrophages (5×10^5^) attached to glass coverslips were infected with GFP-producing *Mtb* (Rv-03, *Mtb* Rv-80lys, Rv-81ami and Rv-82med). The macrophages were fixed, blocked and incubated with H4A3 monoclonal antibodies to LAMP-1, followed by a rhodamine-conjugated goat anti-mouse IgG, as described [38]. Bacterial co-localization with LAMP-1-positive vesicles appeared as yellow spots. The experiments were done in duplicate, and representative images are shown.

### Cytokine measurement in MDMO

Peripheral blood mononuclear cells (PBMC) were isolated from healthy volunteers by differential gradient centrifugation on Ficoll-Paque Plus (Amersham Biosciences). Adherent monocytes were isolated by seeding 5×10^6^ cells in 24-well plates in MDMO-media (RPMI 1640 supplemented with 10% heat-inactivated human serum) and incubating for 90 min at 37°C in 5% CO_2_. Following the removal of non-adherent cells, MDMO-media was added, and cells were incubated at 37°C for 4 days to mature into macrophages and then used for infection with *Mtb* strains. 5×10^5^ macrophages were infected in triplicate in 24-well plates at a multiplicity of infection of 1∶5 as described previously [38]. At indicated periods of infection, supernatants were removed, and the TNF-α and IL-6 levels were measured using ELISA assays (eBioscience, Inc., CA) according to the manufacturer's instructions. In some experiments, MDMO were stimulated with 150 nM PMA, and the secretion of TNF-α was measured.

### Isolation of mycobacterial lipids

The extraction of total lipids from whole cells using chloroform∶methanol (2∶1 v/v) and the separation of polar lipids in a solvent system containing chloroform∶methanol∶water (65∶25∶4) in the first dimension and chloroform∶methanol∶acetic acid∶water (80∶12∶16∶4) in the second dimension were performed as described previously [7],[32],[39]. Polar lipids were visualized by exposing the plates to iodine vapors or staining them with ninhydrin in order to detect amino acid-containing lipids. In some experiments, autoradiography was used to detect radiolabeled lipids. For PDIMs analysis hexane∶diethylether∶acetic acid (80∶20∶1, vol/vol/vol) solvent system was used.

### MALDI-MS, ESI-MS and ESI-MS/MS

MALDI-MS was performed using an UltraFlex TOF/TOF (Bruker Daltonics, Billurica, CA) as described previously [39]. The L-PG sample in acetonitrile was mixed 1∶1 with 2,5-dihydroxylbenzoic acid matrix for spotting onto the target plate.

### Fatty acid analysis

The L-PG was hydrolyzed with 3 N HCl in methanol for 4 h at 80°C. The sample was dried and treated with silylation reagent (TRI-SIL, Pierce Biotechnology, Rockford, IL) for 30 min at room temperature. The trimethylsilylated derivatives were analyzed by GC/MS. Specifically, the sample was applied to a DB-5 column at an initial temperature of 60°C for 1 min, then increased to 130°C at a rate of 30°C/min, and finally increased to 280°C at a rate of 5°C/min.

### NMR spectroscopy

^1^H and ^31^PNMR were performed at a concentration of 2 mg L-PG sample per 0.6 mL of CDCl_3_ on a Varian Inova 400 MHz instrument.

### Amino acid analysis

The purified L-PG was incubated overnight at 100°C in 6 N HCl in a heat-block. Samples were cooled, evaporated to dryness, resuspended in water and subjected to amino acid analysis.

### Neutral red assay

Neutral red chemical staining of *Mtb* wild type and *lysX* mutants was carried out following the protocol described by Soto et al. [40].

### Experiments evaluating the *lysX* phenotype

In order to evaluate the growth inhibitory effects of cationic compounds, Van (1 ug/mL), PMB (100 units/uL), human neutrophil peptide-1 (25 ug/mL) or lysozyme (0.5 mg/mL) was added to the growth media. The human neutrophil peptide stock was dissolved in 0.1% tri-fluoro acetic acid (TFA), and the cultures contained 0.025% TFA. No growth inhibition was noted at this concentration of TFA. The cultures were initially diluted to an OD_600_ of 0.05, dispensed into a 96-well microplate (100 uL per well) and incubated at 37°C with rotation at 60 rpm. At the indicated time periods, the change in the optical density (A_600_) was measured, and viability was determined. Low dose aerosol infection experiments of mice (C57BL/6 female mice) and guinea pigs (Hartely strains) for evaluating the growth and viability of *lysX* strain were essentially as described previously [41].

### Membrane potential analysis

Cytoplasmic membrane potential changes were determined using the slow response, membrane potential-sensitive cyanine dye DiOC_2_(3) (Sigma). Briefly, actively growing cultures of *Mtb* strains (OD_600_ = 0.8) were incubated with 3 µM DiOC_2_(3) for 5 h. Spectrofluorometry was used to detect the red fluorescence (488 nm/620 nm) associated with aggregates of DiOC_2_(3), which exhibits green fluorescence (488 nm/520 nm) in the monomeric state. The assay was performed using white 96-well microtiter plates (Perkin Elmer; Waltham, MA) and a Cary Eclipse spectrofluorometer (Varian; Palo Alto, CA). A negative (depolarized) control of 100 µM *m*-chlorophenylhydrazone CCCP (Sigma), a proton ionophore that destroys the proton gradient and eliminates the bacterial membrane potential, was included. The membrane potential was measured as the ratio of red fluorescence (associated with membrane potential changes) to green fluorescence (a cell size-dependent, membrane potential-independent signal). Preliminary optimization studies revealed that 5 h incubation was optimal for the measurements.

### Statistical analysis

Differences between groups were analyzed by multiple comparison procedures (Student-Newman-Keuls Method) with a simple one-way ANOVA or Mann Whitney Rank Sum Test, using SIGMASTAT (SPSS Science, Inc., Chicago, IL). A *P* value of less than 0.05 was considered significant.

## Supporting Information

Figure S1 Viability of *Mtb* strains in the absence -A- and presence -B- of Van 1.0 µg/ml. At day six of growth, viability was determined by plating cells on Middlebrook 7H11 agar and counting. The bars represent mean±standard error.
(1.57 MB TIF)

Figure S2 Visualization of Fl-Van stained cells. Actively growing cultures (optical density ∼0.01 to 0.04) were grown with fluorescent vancomycin BODIPY (Invitrogen) at a final concentration of 1 µg/mL for 20 hours. The cells were harvested by low speed centrifugation, fixed in 4% paraformaldehyde for 24 h, and imaged on a Nikon Eclipse microscope with a CCD camera; bright field (BF) and fluorescent images (Van) were acquired. Magnification was 100×, and data were analyzed using Metamorph software. At least 100 cells were imaged for each strain; the images were scored for staining patterns and the presence or absence of fluorescent staining. Approximately 35% of wild type and 25% of *lysX* cells were not stained under these conditions. For clarity, only select staining patterns are shown: defined dye accumulation at the cell poles -A, D- and mid-cell (as in D) or diffuse accumulation throughout the cell - B, E. In some cells, both types of accumulation were observed -C, F. For the *lysX* mutant, about 52% of cells showed diffuse staining, but this was only seen in about 32% of wild type cells.
(2.97 MB TIF)

Figure S3 Effect of SDS on the viability of the *lysX* mutant: The viability of *Mtb* strains was examined under the same conditions used for macrophage infection and lysis in the presence of 0.09% SDS. Actively growing cultures of *Mtb* strains were harvested, exposed to 0.09% SDS for 3 min, diluted and spread on Middlebrook 7H11 agar plates. Cells untreated with SDS were processed similarly. All plates were incubated at 37°C, and colonies were counted. No statistically significant differences between the SDS treated and untreated groups were noted. Data shown are mean ± standard error.
(1.05 MB TIF)

Figure S4 Growth of the *lysX* mutant in the spleen of mice and guinea pigs. Growth of the *lysX* mutant and Rv-03 strain in the spleens of mice -A- and guinea pig -B. Following aerosol infection of mice and guinea pigs, spleens were harvested at the indicated time points. Homogenates were prepared, and viability was determined on agar plates. The *lysX* mutant showed reduced and/or delayed dissemination compared to wild type in both animal models.
(1.64 MB TIF)

Figure S5 Neutral red staining and PDIM analysis: Panel A: Wild type and *lysX* mutant cells were stained with neutral red and photographed. As a control, the attenuated strain *Mtb* H37ra was used. Panel B: The lipids were separated by silica thin-layer chromatography (TLC) with hexane:diethylether:acetic acid (80∶20∶1, vol/vol/vol) as solvent system. The lipids were visualized by spraying with 10% phosphormolybdate in ethanol followed by heating to about 110°C for 15 min.
(5.65 MB TIF)
