# Supplementary figures and images for: The Two-Domain LysX Protein of Mycobacterium tuberculosis Is Required for Production of Lysinylated Phosphatidylglycerol and Resistance to Cationic Antimicrobial Peptides
Source: PLoS Pathog. 2009 Jul 31;5(7):e1000534. doi: 10.1371/journal.ppat.1000534 (PMC2713425; doi:10.1371/journal.ppat.1000534)

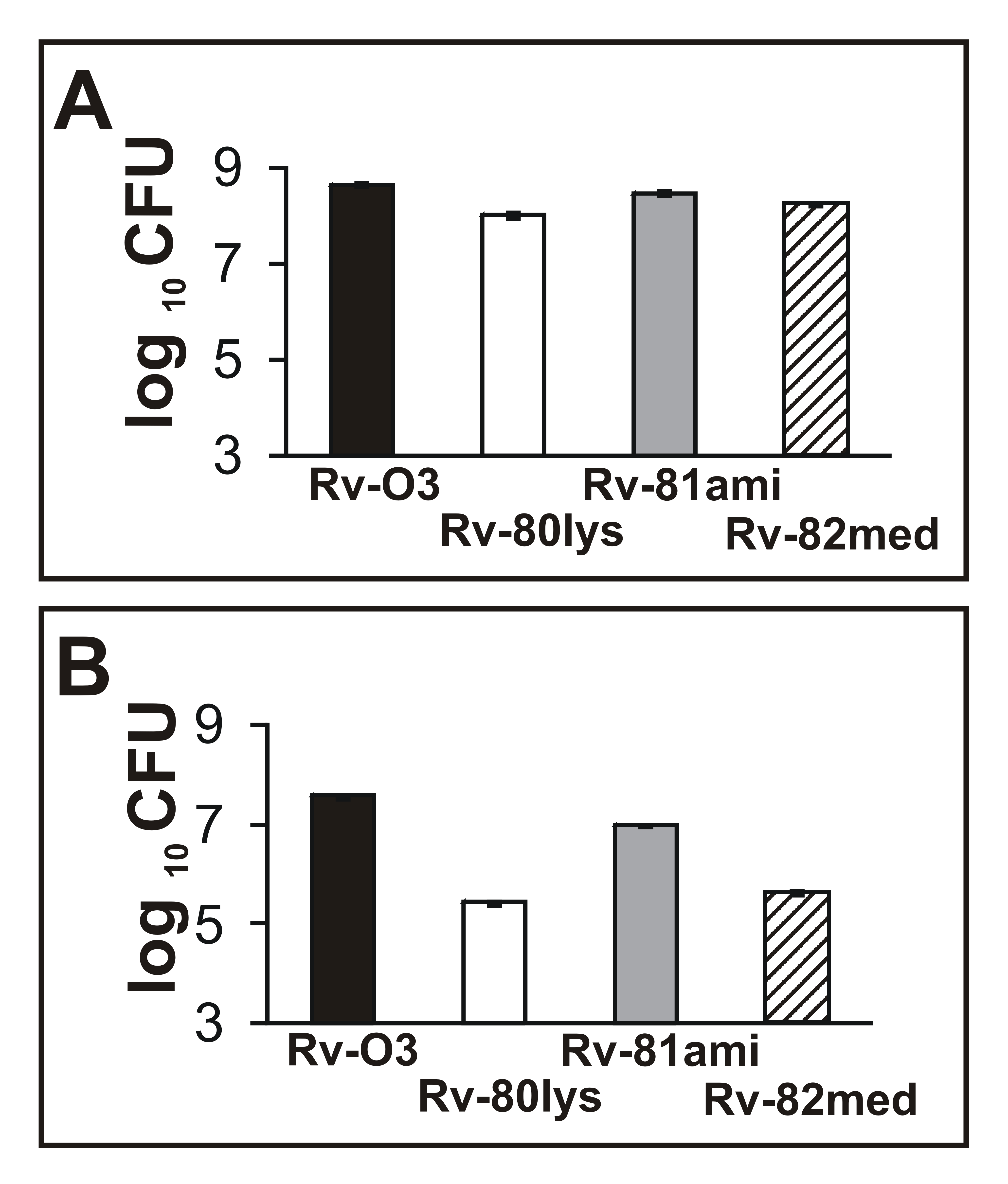

Supplement: Figure S1 — Viability of Mtb strains in the absence -A- and presence -B- of Van 1.0 µg/ml. At day six of growth, viability was determined by plating cells on Middlebrook 7H11 agar and counting. The bars represent mean±standard error. (1.57 MB TIF) [file ppat.1000534.s001.tif]

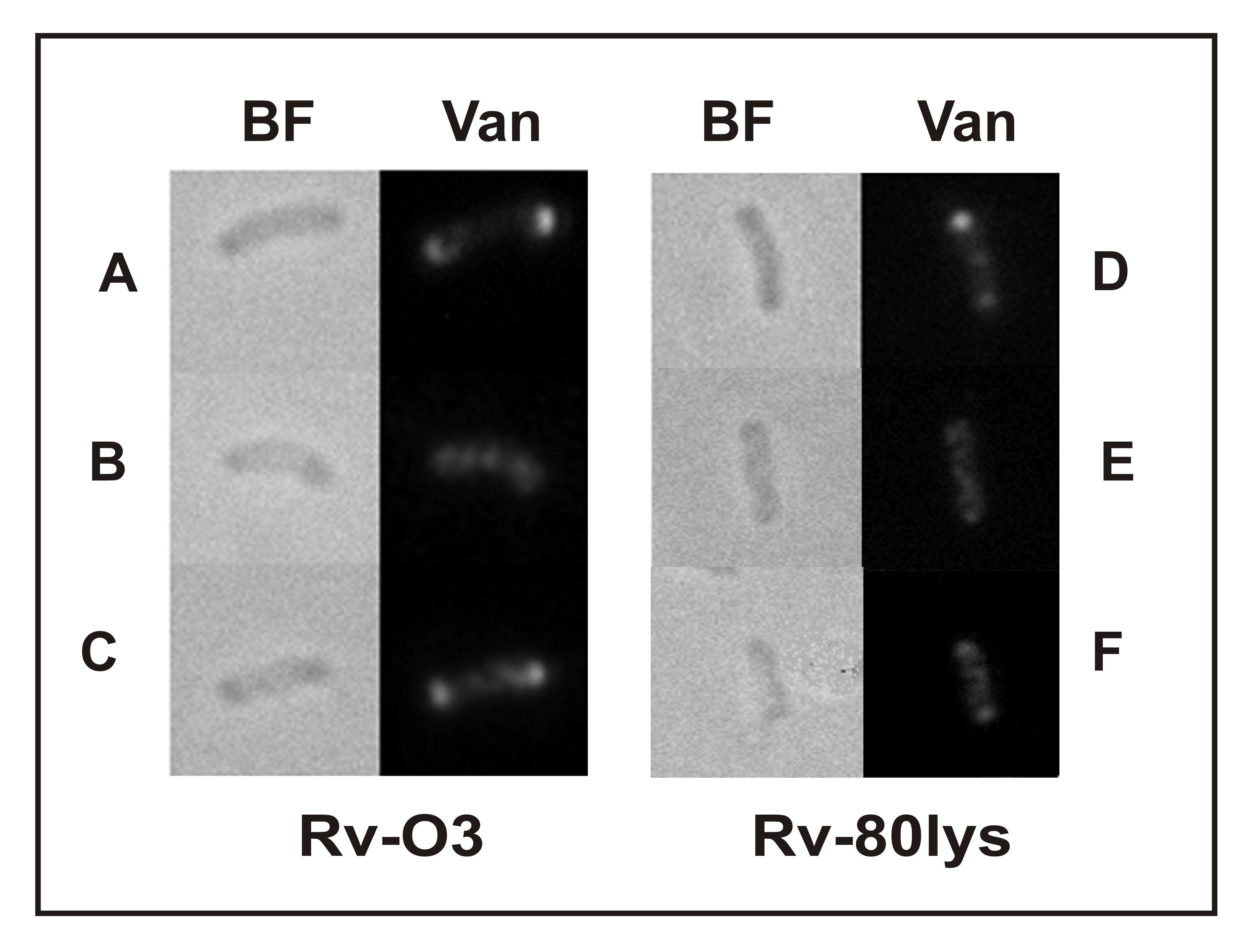

Supplement: Figure S2 — Visualization of Fl-Van stained cells. Actively growing cultures (optical density ∼0.01 to 0.04) were grown with fluorescent vancomycin BODIPY (Invitrogen) at a final concentration of 1 µg/mL for 20 hours. The cells were harvested by low speed centrifugation, fixed in 4% paraformaldehyde for 24 h, and imaged on a Nikon Eclipse microscope with a CCD camera; bright field (BF) and fluorescent images (Van) were acquired. Magnification was 100×, and data were analyzed using Metamorph software. At least 100 cells were imaged for each strain; the images were scored for staining patterns and the presence or absence of fluorescent staining. Approximately 35% of wild type and 25% of lysX cells were not stained under these conditions. For clarity, only select staining patterns are shown: defined dye accumulation at the cell poles -A, D- and mid-cell (as in D) or diffuse accumulation throughout the cell - B, E. In some cells, both types of accumulation were observed -C, F. For the lysX mutant, about 52% of cells showed diffuse staining, but this was only seen in about 32% of wild type cells. (2.97 MB TIF) [file ppat.1000534.s002.tif]

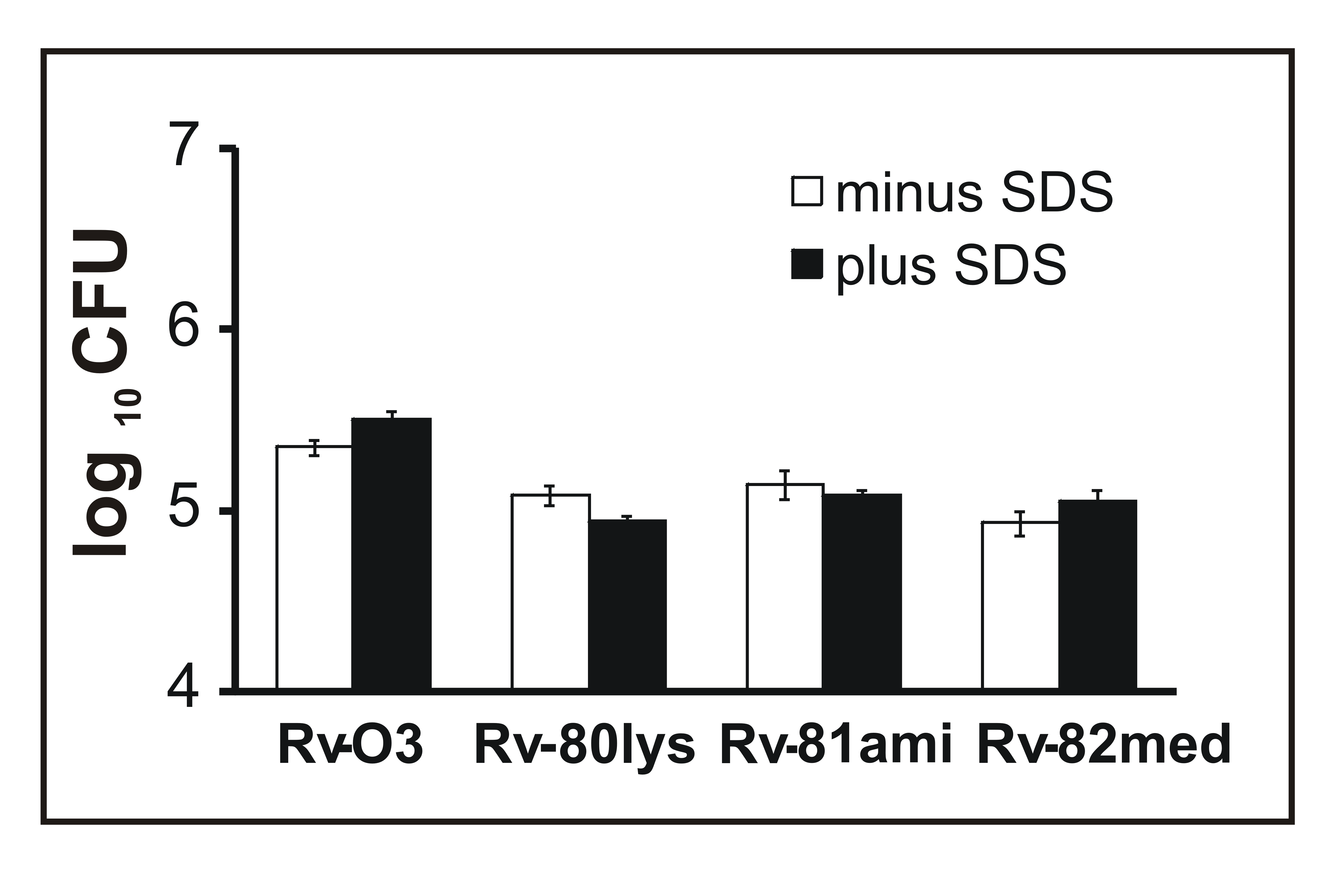

Supplement: Figure S3 — Effect of SDS on the viability of the lysX mutant: The viability of Mtb strains was examined under the same conditions used for macrophage infection and lysis in the presence of 0.09% SDS. Actively growing cultures of Mtb strains were harvested, exposed to 0.09% SDS for 3 min, diluted and spread on Middlebrook 7H11 agar plates. Cells untreated with SDS were processed similarly. All plates were incubated at 37°C, and colonies were counted. No statistically significant differences between the SDS treated and untreated groups were noted. Data shown are mean ± standard error. (1.05 MB TIF) [file ppat.1000534.s003.tif]

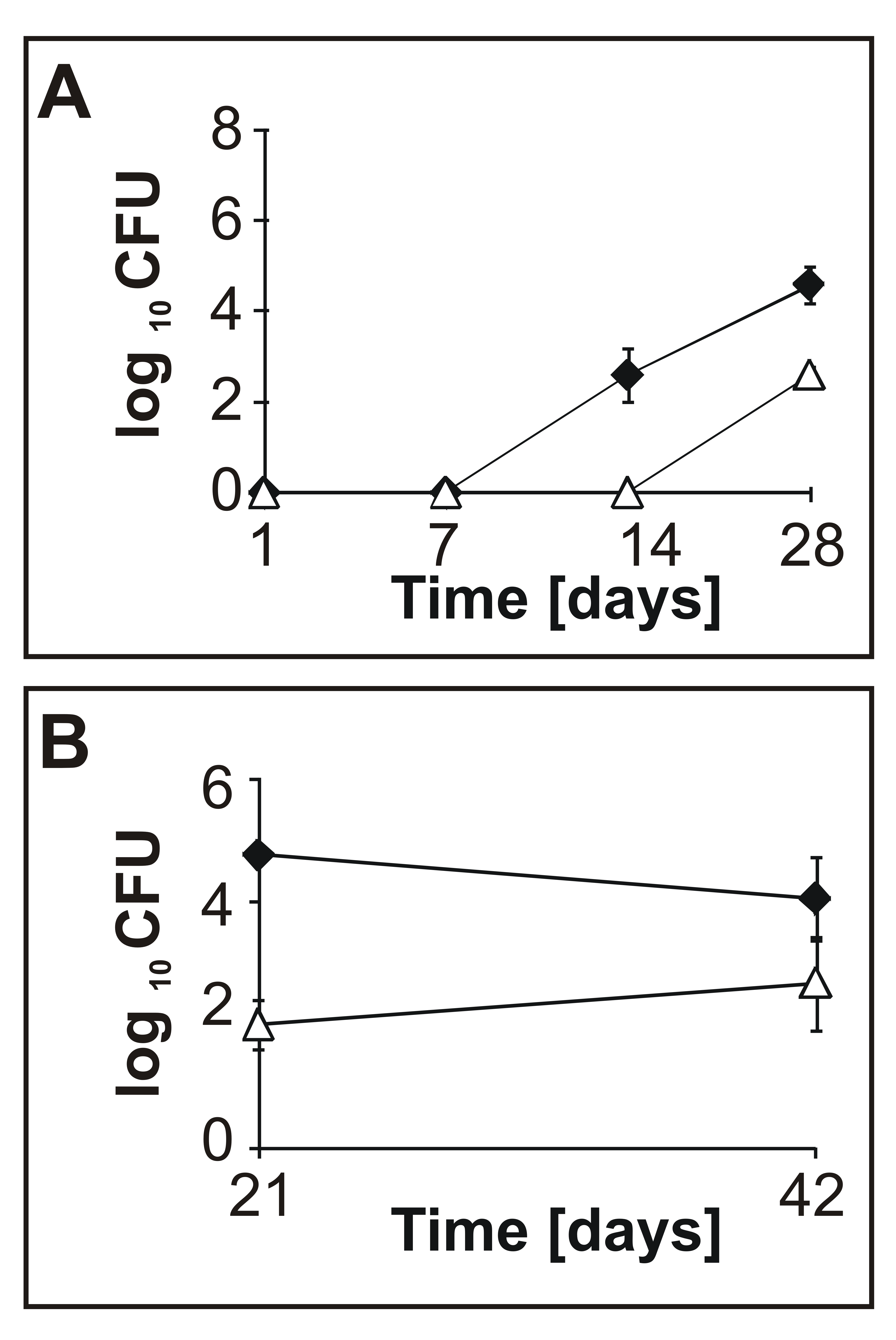

Supplement: Figure S4 — Growth of the lysX mutant in the spleen of mice and guinea pigs. Growth of the lysX mutant and Rv-03 strain in the spleens of mice -A- and guinea pig -B. Following aerosol infection of mice and guinea pigs, spleens were harvested at the indicated time points. Homogenates were prepared, and viability was determined on agar plates. The lysX mutant showed reduced and/or delayed dissemination compared to wild type in both animal models. (1.64 MB TIF) [file ppat.1000534.s004.tif]

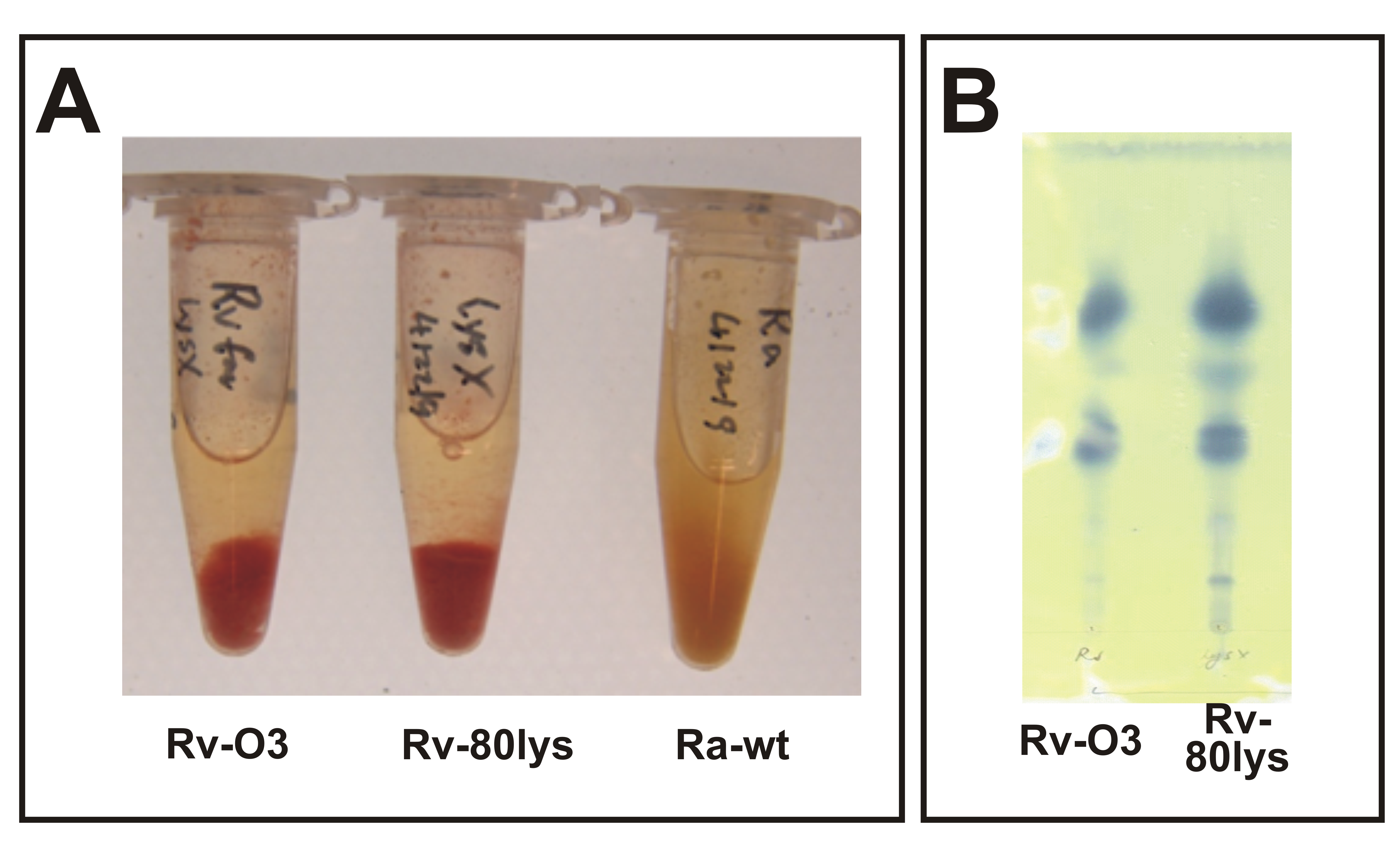

Supplement: Figure S5 — Neutral red staining and PDIM analysis: Panel A: Wild type and lysX mutant cells were stained with neutral red and photographed. As a control, the attenuated strain Mtb H37ra was used. Panel B: The lipids were separated by silica thin-layer chromatography (TLC) with hexane:diethylether:acetic acid (80∶20∶1, vol/vol/vol) as solvent system. The lipids were visualized by spraying with 10% phosphormolybdate in ethanol followed by heating to about 110°C for 15 min. (5.65 MB TIF) [file ppat.1000534.s005.tif]
